# The Role of Identity Motives on Quality of Life and Depressive Symptoms: A Comparison Between Young Adults With Multiple Sclerosis and Healthy Peers

**DOI:** 10.3389/fpsyg.2020.589815

**Published:** 2020-11-16

**Authors:** Emanuela Calandri, Federica Graziano, Martina Borghi, Silvia Bonino, Elena Cattelino

**Affiliations:** ^1^Department of Psychology, University of Torino, Turin, Italy; ^2^Cosso Foundation, Turin, Italy; ^3^CRESM (Regional Referral Multiple Sclerosis Centre) – “San Luigi Gonzaga” Hospital – Orbassano, Turin, Italy; ^4^Department of Human and Social Sciences, University of Aosta Valley, Aosta, Italy

**Keywords:** identity, young adult, depressive symptoms, quality of life, multiple sclerosis, chronic illness, challenge

## Abstract

The diagnosis of a chronic illness during young adulthood represents a non-normative life transition influencing the identity definition process, as well as the individual psychological adjustment. The study examined if relationships between identity motives (self-esteem, efficacy, continuity, distinctiveness, belonging, and meaning), health-related quality of life, and depressive symptoms differ between healthy young adults and young adults diagnosed with multiple sclerosis (MS). Two hundred one people (101 MS patients and 100 healthy controls), aged 18–35 years, completed a self-report questionnaire. Young adults with MS reported lower health-related quality of life and lower efficacy motive than their healthy peers. Among MS patients, high meaning was related to lower depressive symptoms, whereas high continuity and high belonging were related to higher health-related quality of life than in healthy controls. The study highlights the relevance of identity motives for the adjustment to MS and has implications for psychological interventions with young patients.

## Introduction

Young adulthood is recognized as a period of the life course characterized by specific developmental tasks, such as making choices about study and future job career, gaining independence from parents, redefining friendships, and involving in intimate and committed relationships. This period has been recognized as typical of industrialized Western societies, and it is characterized by an individual prolonged exploration of different possibilities, before taking on a complete adult role (Arnett, 2000; Smorti et al., 2020). For some decades, developmental psychology (e.g., Bronfenbrenner, 1979; Baltes, 1997; Elder and Shanahan, 2006; Magnusson and Stattin, 2006) has highlighted that development takes place across the entire life cycle. Moreover, in the process of mutual interaction between the individual and the context, the former has a central and active role. Developmental psychology literature has also stressed that development is promoted by challenges or crisis. When challenges are successfully solved, they can lead to development; on the contrary, if they are not overcome, they can limit the ability to cope with future challenges. Success and failure in overcoming challenges are related to constraints and resources. In particular, resources are personal (such as basic dispositions and skills), structural (such as place of living and income), and social (such as size and quality of social networks). In their lifespan developmental model, Hendry and Kloep (2002) have clearly highlighted the relationships between resources, challenges, and risks: when the individual resources easily match the challenge, the task can be considered as a routine; when challenges exceed the resources available to the individual, they represent a risk for development; when individuals are successful in facing a challenge and their resources are transformed and increased, development has occurred.

Within this theoretical framework, the present study analyzes a particular challenge associated with a non-normative, unpredictable event that occurs only for few individuals, that is, a chronic illness during young adulthood. The diagnosis of a chronic illness during young adulthood can be a risk factor for future development because it introduces numerous constraints in people’s lives and can reduce their resource system. Young people diagnosed with a chronic illness have to face a double challenge, linked to the specific and normative transition of this period of life, as well as to the negative and lifelong event represented by the illness, which requires an active and demanding process of adjustment by the individual. It is therefore important to understand which aspects are involved in this process.

In the present study, we focus on the construct of identity and examine its role with respect to adjustment among young adults with and without a chronic illness. Identity has been defined as the sense of uniqueness and continuity over time that everyone experiences during one’s life despite the continuous changes in biological, psychological, and social aspects of life (Bosma and Kunnen, 2001). The identity exploration is one of the distinctive features of young adulthood (Arnett, 2000), and the identity definition is involved in all the developmental tasks of this age. Identity is considered one of the core predictors of young people’s successful adjustment, along with parent and peer relationships (Schwartz, 2016). In particular, the ability of young adults to develop a coherent sense of identity in different domains has proven to be associated with greater well-being, lower levels of internalizing symptoms, and lower involvement in risk behavior (Schwartz et al., 2011, 2015; Meca et al., 2015). While most of the research traditionally examined the adaptive role of identity in the normative life transitions of young adulthood, the role of identity among young adults suffering from a chronic illness is underinvestigated in literature (Schwartz et al., 2013). In particular, the diagnosis of a chronic illness during young adulthood represents an unexpected break during the process of identity definition (Charmaz, 1983). To our knowledge, existing literature on identity among young adults with chronic health problems mainly focused on diabetes (Pacaud et al., 2007; Luyckx et al., 2008) and congenital heart disease (Luyckx et al., 2011). Results of these studies suggested that a coherent and well-organized sense of identity is related to a better illness-related coping. Moreover, identity can represent a resource in providing meaning and a sense of purpose in the lives of young people suffering from a chronic illness and can protect them against emotional difficulties (Luyckx et al., 2008, 2011; Schwartz et al., 2013). The present study was aimed at extending this area of research focusing on the role of identity for the adjustment to multiple sclerosis (MS), a chronic neurological disease not considered in previous studies on identity and chronic illness among young adults. MS is usually diagnosed to people between 20 and 30 years of age, and it is prevalent among women (the ratio is 3:1 compared to men) (Sellner et al., 2011). Currently, MS diagnoses are made earlier than before, and MS is the most common cause of nontraumatic disability in young adults (Vanbellingen and Kamm, 2016). MS symptoms are manifold (fatigue, pain, motor and sensory deficits, bladder, bowel, and sexual disturbances, and cognitive impairment). The most common form of MS is the RR-MS (relapsing–remitting multiple sclerosis) characterized by relapses alternating with periods of symptom remission, whereas for a minority of patients, the course is characterized by worsening of symptoms and disability from the onset without remissions (primary progressive multiple sclerosis, PP-MS) (Lublin et al., 2014; Calandri et al., 2019). Drugs are used to treat attacks and slow down the disease, but at present, there is no cure for it. The characteristics of MS (chronicity, unpredictable course, multiple and fluctuating symptoms over time) have significant psychological effects on patients (Calandri et al., 2019). In particular, MS has a deep impact on the sense of personal identity because the illness affects the self-image and the body functioning, and requires the individual to modify life plans, chances of personal realization, and social roles (Weinreich and Saunderson, 2003). Young people diagnosed with MS suddenly perceive themselves as ill and have to face the double challenge of redefining their identity as young adults and as people with a chronic illness. This means to reorganize their future perspectives and their life goals, taking into account limits and possibilities linked to the illness, and elaborating a self-image that includes the illness, and does not merely coincide with it (Bonino, 2020). Previous studies showed that successfully redefining one’s identity is related to lower depressive symptoms and higher quality of life and psychological well-being among MS patients (Irvine et al., 2009; Mozo-Dutton et al., 2012; Tabuteau-Harrison et al., 2016; Calandri et al., 2017a, 2019; Rahn et al., 2017). It is therefore crucial to deepen knowledge about identity, as one of the factors that might promote young adults’ adjustment to MS.

In the present study, the construct of identity was operationalized with reference to the integrative model proposed by Vignoles and colleagues (Vignoles et al., 2006; Vignoles, 2011) (Motivated Identity Construction Theory, MICT) that appears particularly suitable for studying identity in a situation of chronic illness. According to this model, identity definition is guided by six fundamental psychological motives: self-esteem, efficacy, continuity, distinctiveness, belonging, and meaning. People are motivated to see themselves in a positive way (the self-esteem motive); to feel competent and capable of influencing their environment (the efficacy motive); to preserve a sense of continuity over time, despite significant life changes (the continuity motive); to search for a sense of differentiation from others (the distinctiveness motive); to maintain a sense of closeness and acceptance by other people within their social contexts (the belonging motive); and finally, to perceive that their lives are meaningful (the meaning motive). The influence of these motives on the process of identity construction has been studied in various domains and in different periods of the life cycle, for example, in the representation of future desired self among adolescents and adults (Vignoles et al., 2008), in the process of identification with groups among young adults (Vignoles and Moncaster, 2007; Easterbrook and Vignoles, 2012), and during young adults’ life transitions (Manzi et al., 2006). To our knowledge, no previous studies examined the role of identity motives in a situation of chronic illness. The model of Vignoles (Vignoles et al., 2006; Vignoles, 2011) is useful for this purpose because it integrates different identity dimensions that are threatened when people are diagnosed with a chronic illness, in particular the efficacy, continuity, and meaning motives. In fact, MS symptoms and disabilities negatively influence the sense of self-efficacy and personal control over the life events and raise the need to redefine life goals and strategies to reach them (Schmitt et al., 2014; Wilski and Tasiemski, 2016; Bonino et al., 2018; Calandri et al., 2018). Moreover, being diagnosed with MS marks a fracture between the “before” and “after” the diagnosis, and involves the need to rebuild a sense of continuity in one’s life (Charmaz, 1983; Bonino, 2020). Finally, with its unpredictable course and multiple disabilities, MS challenges the individual sense of coherence and purpose in life, and requires an ongoing process of meaning reconstruction in one’s life experience (Antonovsky, 1987; Pakenham, 2007; Calandri et al., 2017b).

### The Present Study

The present study explored the role of identity motives for the adjustment to MS, evaluated through the presence of depressive symptoms and the perceived health-related quality of life, by comparing young adults with MS with healthy controls. In light of the examined literature, the aims of the study were: (1) to describe identity motives, depressive symptoms, and health-related quality of life in a group of young adults (18–35 years of age) diagnosed with MS and to compare them with a healthy control group; (2) to investigate if identity motives play a differential role on depressive symptoms and health-related quality of life among young adults with MS compared to healthy controls. The age range of young adults (18–35 years) was chosen with reference to recent literature on life-span development (Santrock, 2019) and to previous studies highlighting the long transition to adulthood, especially in Mediterranean countries (EUROSTAT, 2019). In particular, in Italy, young people generally gain economic independence, leave parental home, and get involved in committed relationships and parenthood over a long period of time, up to 35 years of age (Crocetti et al., 2012; Crocetti and Meeus, 2014; ISTAT, 2020).

With respect to the first aim, we explored if levels of identity motive satisfaction, depressive symptoms, and health-related quality of life differed between young adults with MS and healthy controls. Studies generally showed that MS patients reported lower quality of life and higher depressive symptoms than healthy subjects (Janssens et al., 2003; Jones et al., 2012; Klevan et al., 2014), but literature on young adult patients is lacking, and sometimes, results are inconsistent. Some studies reported high levels of anxiety (Jones et al., 2012), clinically significant depressive symptoms (Buchanan et al., 2010), and reduced quality of life among young adult MS patients (Rainone et al., 2016), but the only study that compared young adult MS patients to healthy controls did not find differences in levels of depression, anxiety, quality of life, self-esteem, and self-efficacy (Messmer Uccelli et al., 2016). To our knowledge, no previous studies investigated identity motives among young adult MS patients. In the light of this literature, we expected that young adults with MS report lower health-related quality of life and higher depressive symptoms than healthy controls, whereas no hypothesis was formulated with respect to differences in identity motives.

As for the second aim, we explored if the condition of chronic illness represents a moderator of the relationships between identity motives, on the one hand, and depressive symptoms and health-related quality of life, on the other hand. As previously said, the diagnosis of a chronic illness affects the sense of personal control over the life events (Schmitt et al., 2014; Wilski and Tasiemski, 2016; Bonino et al., 2018; Calandri et al., 2018), represents a break from past to present (Charmaz, 1983), and raises the need of giving sense to one’s life with the illness (Pakenham, 2007). For this reason, we expect that the efficacy, continuity, and meaning motives would be related to a better adjustment especially among young adults with MS. No hypotheses were formulated in relation to the role of the other identity motives (self-esteem, distinctiveness, and belonging).

## Materials and Methods

### Participants and Procedure

Young adults diagnosed with MS were consecutively recruited at an MS Clinic Center [Regional Referral Multiple Sclerosis Centre (CRESM), Torino, Italy], as part of a larger study on psychological adjustment to MS. The inclusion criteria were: (1) confirmed diagnosis of MS and (2) aged between 18 and 35. The exclusion criteria were: (1) presence of severe psychiatric problems and (2) presence of clinically significant cognitive deficits. All clinical information was retrieved from medical records. Patients who agreed to participate, completed an anonymous self-report questionnaire, administered by a psychologist of the research team, during one of the routine scheduled outpatient visits at the Clinic Center (Calandri et al., 2019). Healthy controls were drawn from the general population. The inclusion criteria were being aged between 18 and 35; the exclusion criteria were being diagnosed with MS or other chronic illness. Healthy control participants were recruited at university courses and in different working and leisure settings. They were administered the same anonymous self-report questionnaire in the recruitment settings by trained researchers. After completion, all the questionnaires (patients and controls) were immediately returned to the researcher in a closed envelope.

The study was approved by the Hospital Ethics Committee (protocol number 0013772), and written informed consent was obtained from all individual participants included in the study. No benefit was given to participants for taking part in the research.

### Measures

*Identity motives* were evaluated using the Identity Motives Scale (Manzi et al., 2010), which considers the six identity motives of the Motivated Identity Construction Theory (self-esteem, efficacy, continuity, belonging, distinctiveness, and meaning). Each motive is represented by two items, one positive (e.g., “When I think about my future, I think I will feel proud,” satisfaction for self-esteem) and the other negative (e.g., “When I think about my future, I think I will feel powerless,” threat to self-esteem). Each item ranges from 1 (“extremely disagree”) to 5 (“extremely agree”), and negative items are reverse coded. Higher scores on each subscale represent higher satisfaction of each identity motive (range 2–10) (Cronbach’s alpha for the total scale = 0.75).

*Depressive symptoms* were assessed through the Italian validation of the 10-item Center for Epidemiologic Studies Depression Scale (CES-D-10, Fava, 1983), which evaluates the frequency of depressive symptoms during the past week (e.g., “I was bothered by things that usually don’t bother me”). Each item is scored on a four-point scale ranging from 0 (“rarely or none of the time”) to 3 (“most or all of the time”). The scale ranges from 0 to 30, with a cutoff score of 10 or higher indicating the presence of significant depressive symptoms. Cronbach’s alpha in our study was 0.85.

*Health-related quality of life* was assessed using the Italian version of the SF-12 Health Survey (Apolone et al., 2005), which is the short version of the SF-36 and represents a validated and widely used self-report instrument assessing health status, both in clinical and healthy samples. It is composed of 12 items, which provide measures of Physical Health (PCS-12) (e.g., “Does your health limit you in climbing one flight of stairs?”; the range of responses include “Yes, limited a lot,” “Yes, limited a little,” and “No, not limited at all”) and Mental Health (MCS-12) (e.g., “How much of the time during the past 4 weeks did you felt calm and peaceful?”; responses ranged from 1 = “all of the time” to 6 = “none of the time”). For each scale, the standardized scores ranged from 0 to 100 (mean score = 50; *SD* = 10). In the present study, we considered the sum of PCS-12 and MCS-12 as a measure of overall health-related quality of life (Apolone et al., 2005) (Cronbach’s alpha = 0.86).

### Statistical Analyses

A preliminary check on missing data was carried out, and the percentage of missing response was less than 10%. The MCAR (Missing Completely at Random) test (Little, 1988) showed nonsignificant results for depressive symptoms and health-related quality of life (missing data were imputed with the Expectation–Maximization procedure) and significant results for identity motives (missing data were imputed through the Regression procedure). Preliminary descriptive analyses included *t*-tests for differences in study variables between patients and healthy controls, Cohen’s d as a measure of *t*-test effect size, and Pearson’s bivariate correlations. Then, according to the aims of the study, we ran two regression models to predict depressive symptoms and health-related quality of life, respectively. Independent variables were gender (1 = men), health/illness condition (1 = people with MS), mean centered identity motives (Aiken and West, 1991), and the interactions between each identity motive and the health/illness condition. To interpret significant interactions, we plotted the effects and performed a simple slope analysis. We tested the effects at low (mean −1 sd) and high (mean +1 sd) levels of the moderator. All statistical analyses were performed with SPSS Statistics 25.

## Results

### Descriptive Statistics

The final convenience sample included 101 MS patients and 100 healthy controls (total 201 participants; 66.7 % women; *M* age = 26.3, sd = 4.3, range 18–35 years). Demographic data are reported in Table 1. Concerning clinical variables in the MS sample, all participants had the relapsing–remitting form of MS, with a mean disease duration of 1.6 years (SD = 0.8; range 1–10) and a mild disability (mean EDSS score = 1, sd = 0.9; range 0–3)^1^. No differences were observed between the two samples (people diagnosed with MS and healthy controls) with regard to gender, *χ^2^*(1) = 0.64, *p* = 0.425, mean age, *t*(199) = −1.88, *p* = 0.062, living situation, *χ^2^*(4) = 9.23, *p* = 0.056, and employment, *χ^2^*(1) = 1.55, *p* = 0.214. A statistically significant difference only emerged for the education level, which was higher among the healthy controls, *χ^2^*(2) = 9.28, *p* < 0.05.

**TABLE 1:** Characteristics of the study participants (*N* = 201).

	Multiple sclerosis (MS) patients (*N* = 101)		Healthy controls (*N* = 100)	
	*N*	%	*N*	%
**Gender^1^**				
Women	70	69	64	64
Men	31	31	36	36
Age (years, *M, sd*)	26.9 (4.2)		25.7 (4.4)	
**Living situation^2^**				
With parents	43	43	45	48
With partner	36	36	17	18
With partner and children	6	6	9	10
Alone	15	15	22	24
**Education**				
At least 8 years (middle school diploma)	15	15	5	5
At least 13 years (high school diploma)	55	54	47	47
More than 13 years (degree)	31	31	48	48
**Employment**				
Employed	71	70	62	62
Unemployed/student	30	30	38	38
**Clinical variables**				
RR-MS (relapsing–remitting multiple sclerosis)	101	100		
Disease duration (years, *M, sd*) (range 1–10)	1.6 (0.8)			
Level of disability (EDSS score)^3^ (*M, sd*) (range 0–3)	1 (0.9)			

*^1^The gender distribution reflects women’s prevalence of MS. ^2^Data were missing for one MS patient and seven healthy controls. ^3^The Expanded Disability Status Scale (EDSS) score (Kurtzke, 1983) is the most widely used measure of disability in MS, and it is evaluated by a neurologist. Patients in our study had mild levels of disability.*

Means and standard deviations of study variables are reported in Table 2. People with MS reported lower health-related quality of life and lower efficacy motive than healthy controls, whereas no statistically significant differences emerged for depressive symptoms and for the other identity motives.

**TABLE 2:** Descriptive statistics of study variables among MS patients and healthy controls (*N* = 201).

	MS patients (*N* = 101)		Healthy controls (*N* = 100)		Student’s *t*_(df)_	*p*	Cohen’s *d*
	*M*	*sd*	*M*	*sd*			
**Identity motives**							
Self-esteem	7.97	1.59	8.10	1.44	−0.61_(199)_	0.545	0.09
Efficacy	6.80	1.66	7.81	1.48	4.54_(199)_	0.0001	0.64
Continuity	6.32	1.91	6.17	1.83	−0.56_(199)_	0.578	0.08
Distinctiveness	7.18	2.01	7.20	1.65	0.08_(199)_	0.933	0.01
Belonging	8.10	1.63	8.39	1.42	1.35_(199)_	0.179	0.19
Meaning	7.60	2.01	7.89	1.66	1.10_(199)_	0.273	0.16
Depressive symptoms	8.80	5.86	8.39	4.64	−0.55_(199)_	0.581	0.08
HRQoL	95.51	14.39	100.24	8.29	2.86_(199)_	0.005	0.40

*HRQoL, health-related quality of life.*

A depressive symptom score equal or greater than 10, representing the critical cutoff for the presence of depressive symptoms, was reported by 42% of MS patients and 35% of healthy controls, but the difference was not statistically significant, *χ^2^*(1) = 0.92, *p* = 0.337.

Results of the correlation analysis are reported in Table 3. Statistically significant negative correlations emerged between all identity motives and depressive symptoms (values ranging from *r* = −0.26 and *r* = −0.56, *p* < 0.01), whereas statistically significant positive correlations were found between all identity motives and health-related quality of life (values ranging from *r* = 0.22 to *r* = 0.44, *p* < 0.01). The identity motives were positively correlated to each other with statistically significant *r*-values ranging from 0.17 to 0.73, *p* < 0.01. Gender was positively correlated to the self-esteem motive (*r* = 0.17, *p* < 0.05) and the efficacy motive (*r* = 0.16, *p* < 0.05), indicating a greater endorsement of these motives among men. Gender was also positively correlated to health-related quality of life (*r* = 0.16, *p* < 0.05), and negatively to depressive symptoms (*r* = −0.19, *p* < 0.01), indicating higher health-related quality of life and lower depressive symptoms among men.

**TABLE 3:** Bivariate correlations among study variables.

		1	2	3	4	5	6	7	8	9	10
1	Gender^1^	–									
2	Age	0.06	–								
3	Self-esteem	0.17*	0.06	–							
4	Efficacy	0.16*	−0.14	0.33**	–						
5	Continuity	−0.03	0.01	0.12	−0.01	–					
6	Distinctiveness	0.13	0.01	0.51**	0.23**	0.04	–				
7	Belonging	−0.01	−0.03	0.45**	0.47**	0.17*	0.23**	–			
8	Meaning	0.11	0.03	0.73**	0.43**	0.18*	0.41**	0.51**	–		
9	Depressive symptoms	−0.19**	−0.11	−0.49**	−0.28**	−0.26**	−0.26**	−0.41**	−0.56**	–	
10	HRQoL	0.16**	−0.05	0.38**	0.26**	0.25**	0.22**	0.31**	0.44**	−0.76**	–

^1^Spearman correlations (0 = women, 1 = men). *p < 0.05 **p < 0.01.

### Predictors of Depressive Symptoms

**FIGURE 1:**
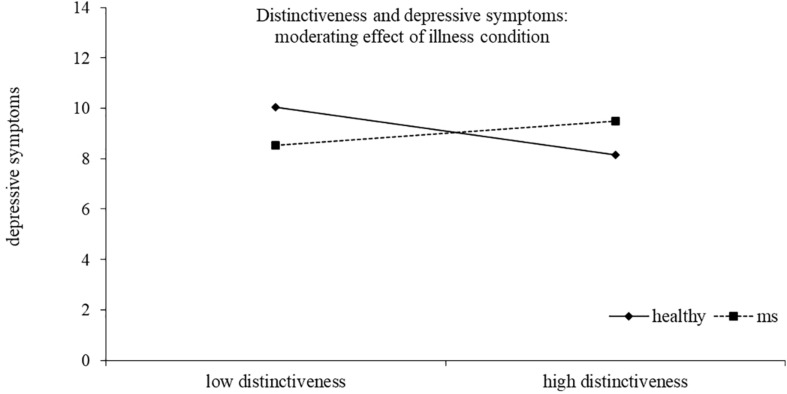
Distinctiveness and depressive symptoms: moderating effect of illness condition.

**FIGURE 2:**
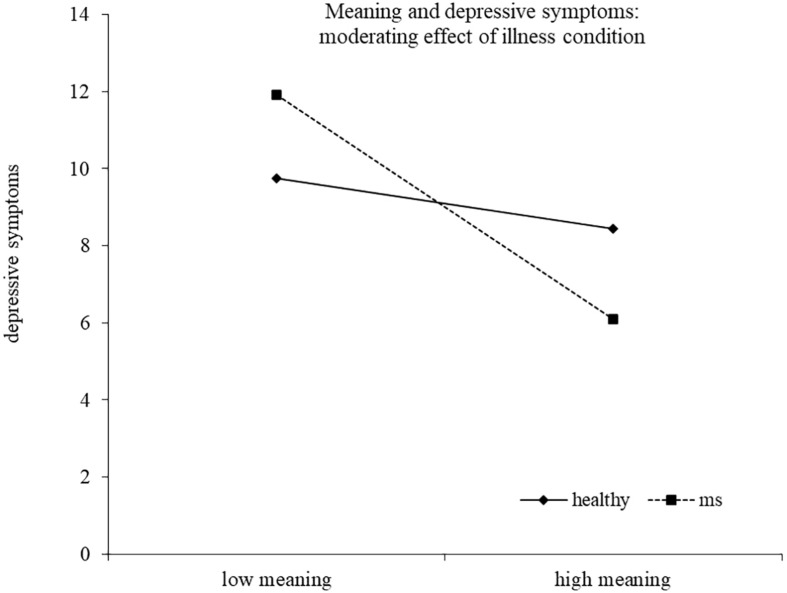
Meaning and depressive symptoms: moderating effect of illness condition.

Results of the regression model are reported in Table 4. The model explained 40% of the variance in depressive symptoms scores, *F*(14,186) = 10.43, *p* < 0.0001^2^. Two interactions emerged as statistically significant, namely, distinctiveness X health/illness condition and meaning X health/illness condition, indicating that the association between these identity motives and depressive symptoms was moderated by the presence of the illness. High distinctiveness tended to be related to lower depressive symptoms for healthy young adults, although the simple slope analysis did not reach statistical significance (healthy *b* = −0.52, *t* = −1.72, *p* = 0.088; MS *b* = 0.26, *t* = 1.07, *p* = 0.286). High meaning was related to lower depressive symptoms only for young adults with MS. The simple slope was in fact significant for MS participants (*b* = −1.58, *t* = −4.67, *p* < 0.0001), but not for healthy controls (*b* = −0.35, *t* = −0.91, *p* = 0.363) (Figures 1, 2).

**TABLE 4:** Predictors of depressive symptoms and health-related quality of life (multiple regression analysis).

	Depressive symptoms				Health-related quality of life			
	*B*	*SE B*	*β*	*p*	*B*	*SE B*	*β*	*p*
Intercept	9.10	0.48		0.000	98.68	1.15		0.000
Healthy/MS^1^	−0.09	0.61	−0.01	0.879	−**3.71**	**1.46**	−**0.16**	**0.012**
Gender^2^	−1.08	0.64	−0.10	0.096	1.65	1.54	0.07	0.283
Self-esteem	−0.71	0.40	−0.20	0.080	−0.05	0.95	−0.01	0.961
Efficacy	−0.46	0.37	−0.15	0.209	**2.14**	**0.88**	**0.29**	**0.016**
Continuity	−0.32	0.23	−0.11	0.174	0.31	0.56	0.05	0.578
Distinctiveness	−0.52	0.30	−0.18	0.088	0.49	0.72	0.08	0.490
Belonging	−0.06	0.36	−0.02	0.879	−1.25	0.86	−0.16	0.147
Meaning	−0.35	0.39	−0.12	0.363	0.63	0.92	0.10	0.493
Self-esteem *X* healthy/MS	0.69	0.61	0.15	0.255	1.83	1.46	0.17	0.211
Efficacy *X* healthy/MS	0.78	0.46	0.18	0.091	−**3.08**	**1.09**	−**0.31**	**0.005**
Continuity *X* healthy/MS	−0.28	0.32	−0.07	0.386	**1.79**	**0.77**	**0.20**	**0.022**
Distinctiveness *X* healthy/MS	**0.78**	**0.39**	**0.21**	**0.046**	−0.74	0.92	−0.09	0.423
Belonging *X* healthy/MS	−0.67	0.48	−0.15	0.167	**2.80**	**1.14**	**0.27**	**0.015**
Meaning *X* healthy/MS	−**1.23**	**0.52**	−**0.33**	**0.018**	1.21	1.23	0.14	0.324

*^1^Healthy/MS (0 = healthy controls, 1 = people with MS). ^2^Gender (0 = women, 1 = men). In bold, statistically significant predictors *p* < 0.05.*

### Predictors of Health-Related Quality of Life

**FIGURE 3:**
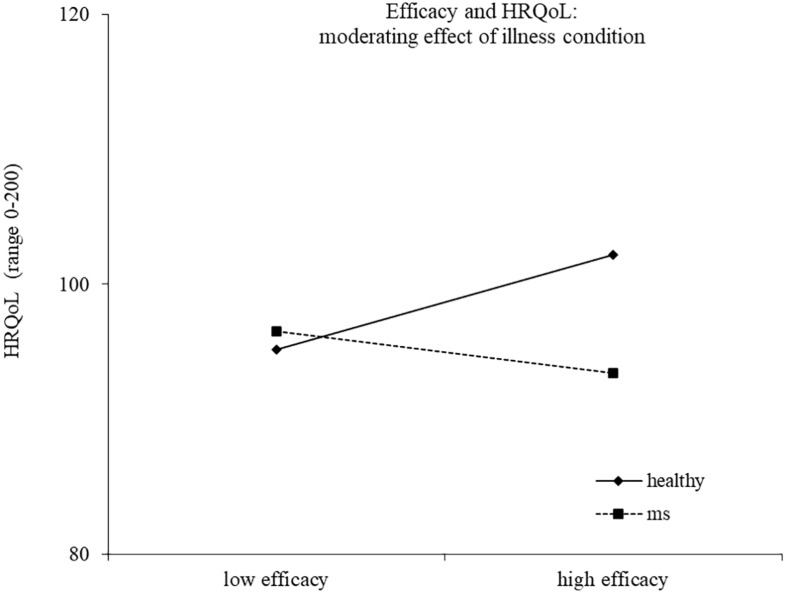
Efficacy and HRQoL: moderating effect of illness condition.

**FIGURE 4:**
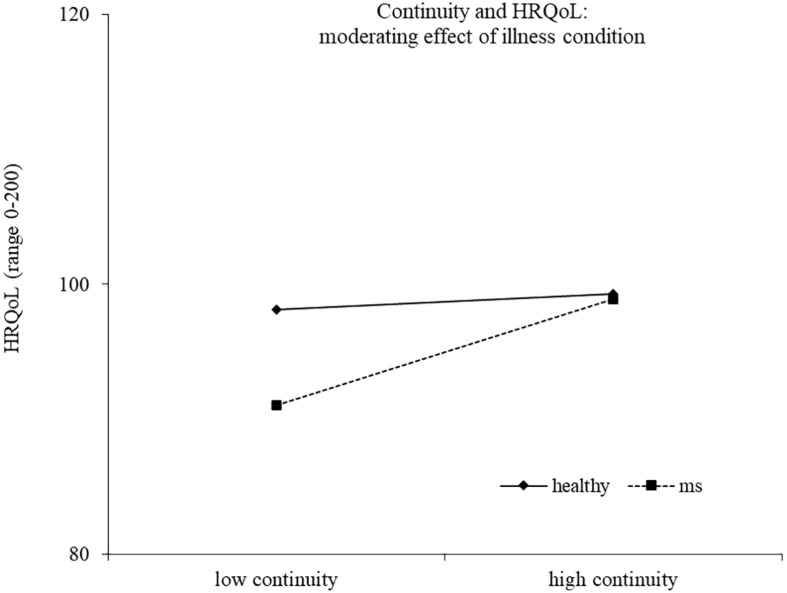
Continuity and HRQoL: moderating effect of illness condition.

**FIGURE 5:**
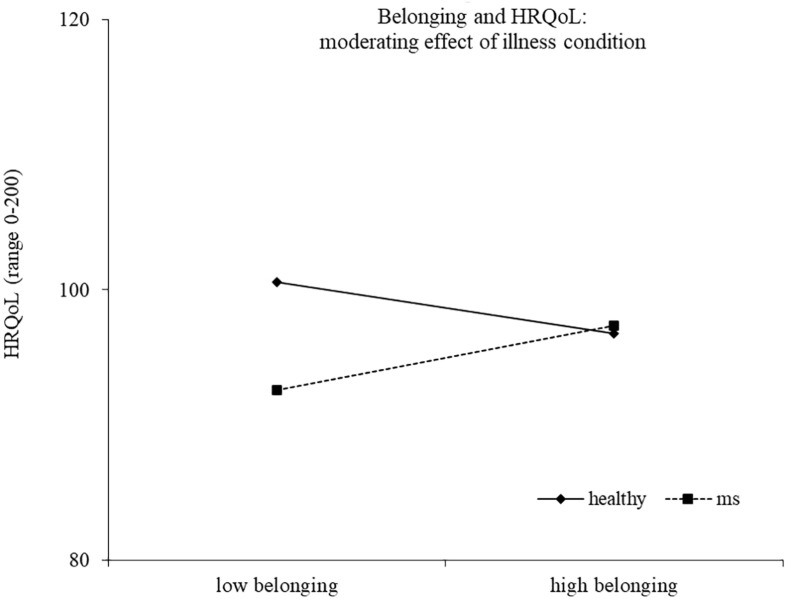
Belonging and HRQoL: moderating effect of illness condition.

Results of the second regression model are reported in Table 4. The model explained 33% of the variance in health-related quality of life, *F*(14,186) = 8.17, *p* < 0.0001. The main effects of health/illness condition and efficacy emerged, indicating a higher health-related quality of life for healthy controls and for people reporting a high efficacy motive, respectively. The effect of efficacy was moderated by the health/illness condition, with high efficacy related to higher health-related quality of life for healthy controls (*b* = 2.14, *t* = 2.44, *p* = 0.016), but not for young with MS (*b* = −0.94, *t* = −1.42, *p* = 0.157) (Figure 3). Two other interactions emerged as statistically significant, namely, continuity X health/illness condition and belonging X health/illness condition, indicating that the association between these two identity motives and health-related quality of life was moderated by the presence of MS. High continuity was related to higher health-related quality of life for young participants with MS (*b* = 2.10, *t* = 3.93, *p* = 0.0001), but not for healthy controls (*b* = 0.31, *t* = 0.56, *p* = 0.578). Similarly, high belonging was related to higher health-related quality of life for young adults with MS (*b* = 1.55, *t* = 2.04, *p* = 0.043), but not for healthy controls (*b* = −1.25, *t* = −1.46, *p* = 0.147) (Figures 4, 5).

## Discussion

The study was aimed at describing identity motives, depressive symptoms, and health-related quality of life in a group of young adults affected by MS compared with healthy controls, and to explore if identity motives play a differential role on depressive symptoms and health-related quality of life among ill and healthy young adults. People with MS reported lower health-related quality of life than healthy controls, whereas the two groups did not differ with respect to depressive symptoms. Concerning differences in identity motives, only efficacy was lower among people with MS than in healthy controls. As hypothesized, the illness condition represents a moderator in the relationship between some identity motives and adjustment. In particular, main results stressed that for young people with MS higher meaning was related to lower depressive symptoms, whereas high continuity and high belonging were related to higher health-related quality of life compared with healthy controls.

### Adjustment and Identity Motives Among Young Adults With MS and Healthy Peers

With regard to the first aim, the result indicating a worse health-related quality of life for young people with MS was consistent with our hypothesis and with previous studies (Janssens et al., 2003; Jones et al., 2012; Klevan et al., 2014). As for the depressive symptoms, we did not find differences between ill and healthy young adults, in line with the study of Messmer Uccelli et al. (2016). This similarity could be explained by the fact that patients in our sample generally had low disability and the presence of depressive symptoms is likely to occur with symptom worsening and illness progression, which pose demanding challenges that are very difficult to face, and even experienced by the individual as insurmountable.

Nonetheless, the preliminary result of our study does not allow reaching definitive conclusions, and further studies are needed with larger and more representative samples. Among the motives, the only difference between ill and healthy young adults concerned efficacy, a dimension clearly influenced by the illness. In fact, physical and psychological symptoms threaten the sense of personal control over life, especially in relation to the limits and the unpredictability of the illness (Schmitt et al., 2014; Wilski and Tasiemski, 2016; Bonino et al., 2018; Calandri et al., 2018).

### Identity Motives and Adjustment: The Moderating Role of the Illness

Some interesting results emerged when considering the moderating role of the illness condition in the relationship between identity motives, depressive symptoms, and health-related quality of life. First of all, as hypothesized, the meaning motive resulted to be relevant with respect to depressive symptoms for young adults with MS. Literature on identity found that the meaning motive is the most negatively influenced in an imaginary situation of perceived threat to identity, (Batory, 2015) and the illness represents a real threat to identity that is supposed to deeply influence the sense of meaningfulness. We found people with MS reporting high meaning experience lower depressive symptoms and the results is in line with studies highlighting that patients who can make sense of their lives with the illness have a better adjustment to MS (Antonovsky, 1987; Pakenham, 2007; Calandri et al., 2017b). This implies to redefine life plans in relation to illness limitations and to elaborate a new self-image that includes the chronic illness, but not merely coincides with it.

As expected, the illness condition represents a moderator also in the relation between the continuity and the health-related quality of life. In particular, our study stressed the relevance of the continuity motive for young people with MS. During young adulthood, individuals experience normative changes and discontinuity in different life domains, like education, work, social, and intimate relationships. The diagnosis of a chronic illness represents a non-normative transition, a deep and unexpected life change, which imposes the need of preserving a sense of self-continuity over time (Charmaz, 1983). This could be the reason for a greater relevance of the continuity motive for young people with MS than for healthy peers. As also found in other studies, the continuity might buffer against threats to identity, and it is associated with positive psychological outcomes (van Doeselaar et al., 2018).

As for the efficacy motive, contrary to our expectations, higher efficacy resulted to be linked to a better health-related quality of life for healthy people, whereas for ill young adults, this relationship was not found. Efficacy is compromised in young people with MS, and therefore, they probably cannot rely on this dimension to face illness difficulties. This result suggests the need to foster the sense of efficacy among MS patients to improve their quality of life (Schmitt et al., 2014; Wilski and Tasiemski, 2016; Bonino et al., 2018; Calandri et al., 2018).

Finally, a third identity motive related to quality of life was belonging. In particular, we found that high belonging was related to a better health-related quality of life for young adults with MS. This result suggests that maintaining a sense of closeness and acceptance by other people is more relevant for the quality of life of ill than healthy young people. The closeness is likely to be linked to the need of social support as a resource to cope with the illness (Lode et al., 2009; Dennison et al., 2011). Moreover, the perception of being accepted by other people might contrast feelings of exclusion and fear of social stigma, and this might be linked to a better quality of life (Dennison et al., 2011; Cook et al., 2016).

### Limitations and Future Directions

The study had some limitations. First, the participants were not representative of the population under study, and this limits the generalizability of the results. Further research involving a larger sample would allow confirmation of the results of the present study. Second, the study was cross-sectional, and relationships between variables cannot be interpreted in terms of causal pathways. A longitudinal research design would allow to deepen knowledge about the protective role of identity motives with respect to adjustment among ill and healthy young people. Third, future research should deepen knowledge about the suggested relationship between the distinctiveness motive and depressive symptoms. Despite the statistical nonsignificance, distinctiveness seemed to be related to lower depressive symptoms only among healthy young adults, suggesting that searching for a sense of differentiation from others might have a different role for ill and healthy youth. In fact, while for healthy people, the sense of distinctiveness may be positive for the self-definition (van Doeselaar et al., 2018), for ill people, the perception of being different might be due to the illness. This perception might reinforce social stigma and feeling of exclusion, and this could lead to increasing depressive symptoms.

## Conclusion

Young adults who receive a diagnosis of MS have to face both the normative transition to adulthood, as their healthy peers, and the non-normative transition to the illness. This challenging situation influences the process of identity redefinition of ill young people, as well as their psychological adjustment. The present study offers a developmental point of view on the study of young adult’s adjustment to a chronic illness, in line with theoretical models that consider non-normative life events like challenging and demanding situations that can lead the individual to a successful development (Hendry and Kloep, 2002). Moreover, the present study adds knowledge to literature on identity motives, focusing on the transition to a condition of illness during young adulthood. Finally, the study expands the literature on psychological adjustment to a chronic illness, focusing on the role of identity that is underinvestigated in relation to MS. Our results suggest that specific identity motives have a differential role for the adjustment of healthy and ill young people. This can help researchers to understand more in depth the transition to young adulthood, offers a better understanding of the experience of illness, and gives useful indications for psychological intervention. The study suggests to clinicians the need of sustaining a process of identity redefinition in different domains when supporting young people diagnosed with MS. In particular, young patients should be supported in finding a meaning in their life with the illness and in preserving a continuity between the “before” and “after” the diagnosis. Moreover, a psychological intervention should be focused on the recovering of a sense of self-efficacy, which is the belief of being able to achieve a goal by carrying out all the necessary steps (Bandura, 1997). Self-efficacy is deeply undermined by a chronic illness, such as multiple sclerosis. Patients should be supported to recover the awareness of having the personal resources to face the challenges posed by the illness, as well as the confidence in one’s ability to mobilize them. In particular, patients should recover a sense of control over life, through strategies that allow them to reach meaningful goals in relation to limits and possibilities of the illness (Bonino, 2020). Finally, interventions should promote strategies to request social support with the final aim of improving the adjustment to the illness.

## Data Availability Statement

The raw data supporting the conclusions of this article will be made available by the authors, without undue reservation.

## Ethics Statement

All procedures performed in studies involving human participants were in accordance with the ethical standards of the institutional and/or national research committee (S. Luigi Gonzaga Hospital (Orbassano, Turin, Italy) Ethics Committee – protocol number 0013772) and with the 1964 Helsinki declaration and its later amendments or comparable ethical standards. The patients/participants provided their written informed consent to participate in this study.

## Author Contributions

ECal provided the study design, interpretation of data, and manuscript writing. FG participated in the study design and coordination, provided statistical analysis, interpretation of the data, and manuscript writing. MB participated in the coordination of the study, recruited participants, and performed the measurement. SB provided the study design, manuscript writing, and project supervision. ECat contributed to the interpretation of the data, recruited participants, and performed the measurement. All authors read and approved the final manuscript.

## Conflict of Interest

The authors declare that the research was conducted in the absence of any commercial or financial relationships that could be construed as a potential conflict of interest.

## Footnotes

1 The Expanded Disability Status Scale (EDSS) score (Kurtzke, 1983) is the most widely used measure of disability in MS, and it is evaluated by a neurologist. Patients in our study had mild levels of disability (0 = no disability; 3 = autonomous, fully ambulatory, mild neurological deficits in three or four functional systems).

2 For both models, colinearity diagnostics showed the absence of multicolinearity between predictors with VIF (variance inflation factor) values for each predictor lower than 7.
